# Immune modulation and cecal microbiome changes in broilers fed with fenugreek seeds and *Bacillus*-based probiotics

**DOI:** 10.1016/j.psj.2024.104130

**Published:** 2024-07-29

**Authors:** Deependra Paneru, Guillermo Tellez-Isaias, Walter G. Bottje, Emmanuel Asiamah, Ahmed A.A. Abdel-Wareth, Md Salahuddin, Jayant Lohakare

**Affiliations:** ⁎ Department of Poultry Science, University of Georgia, Athens, GA 30602, USA; † Center of Excellence in Poultry Science, University of Arkansas, Fayetteville, AR 72701, USA; ‡ Department of Agriculture, University of Arkansas at Pine Bluff, Pine Bluff, AR 71601, USA; § Department of Animal and Poultry Production, Faculty of Agriculture, South Valley University, Qena 83523, Egypt; ǁ Poultry Center, Cooperative Agricultural Research Center, Prairie View A&M University, Prairie View, TX 77446, USA

**Keywords:** broiler, fenugreek, direct-fed microbial, immunity, cecal microbiome

## Abstract

Intensive broiler production systems face challenges like enteric diseases, impacting global food security. Strategies to enhance broiler immunity and gut health, particularly amidst antibiotic growth promoter restrictions, are crucial. The present study investigated the combined effects of fenugreek seeds (**FS**) and *Bacillus*-based direct-fed microbials (**DFM**) on immune-related gene expression in the ileum and alteration of microbial population in the cecum of broiler. The study involved 160 Ross 308 broiler chicks, which were divided into four groups consisting of 5 replicates, each containing eight birds. The chicks were grown for a period of 42 d, during which they had ad libitum access to feed and water. Dietary treatments were: Control (basal diet), FS5 (basal + 5g/kg fenugreek seeds), FS5DFM (basal + 5g/kg fenugreek seeds + 0.1g/kg Bacillus-based DFM), and DFM (basal + 0.1g/kg Bacillus-based DFM). Ileum tissue and cecal contents were collected on d 42 for gene expression and gut microbiome analysis. Ileal gene expression analysis revealed the downregulation of IL-6, IL-8L2, CASP6, PTGS2, and IRF7 in both FSs and DFMs groups compared to the control, suggesting individual immunomodulatory effects. However, avian β-defensin genes exhibited complex regulation, highlighting the need for further investigation. Cecal microbiome diversity remained stable, with subtle shifts in specific taxa influenced by FSs and DFMs. Interestingly, the combination of the FSs and DFMs uniquely impacted specific taxa, including *Clostridiales vadin* BB60. These findings suggest that both FSs and DFMs demonstrated potential for improving broiler immunity through inflammation reduction. The combination of FSs and DFMs offers a synergistic effect in immune modulation and specific microbial modulation, warranting further investigation with pathogen challenge models for comprehensive understanding.

## INTRODUCTION

Broiler chickens, bred specifically for meat production, play a crucial role in global food security. They are an efficient source of high-quality animal protein, readily available and affordable for millions worldwide (Shahbandeh, 2023). However, the intensive production systems employed to meet this demand present various challenges that can negatively impact the health and well-being of these birds, consequently affecting their productivity and raising ethical concerns (Clark et al., 2019). One of these challenges is the prevalence of enteric diseases, such as necrotic enteritis, colibacillosis, and coccidiosis, in commercial poultry, which lead to reduced growth, increased mortality, and impaired feed efficiency (Dziva et al., 2008; M'Sadeq et al., 2015; Blake et al.,2020). Necrotic enteritis alone causes an economic loss of an estimated $6 billion annually to the global poultry industry, with colibacillosis and coccidiosis also contributing to a significant number (Ebrahimi-Nik et al., 2018; Blake et al., 2020; Emami and Dalloul, 2021). Enteric diseases can cause imbalance of the intestinal microbiota, which can result in dysbiosis, inflammation, and susceptibility to other pathogens (Bortoluzzi et al., 2019; Mohebodini et el., 2019; Choi et al., 2023). Therefore, there is a need to find effective and sustainable strategies to improve the immunological response and cecal microbiome of broilers, especially in the context of the ban or restriction of antibiotic growth promoters (**AGP**) in many countries.

One potential strategy is the use of fenugreek seeds (**FS**), which are the seeds of *Trigonella foenum-graecum*, a leguminous plant that has been used as a medicinal herb and a spice for centuries (Ruwali et al., 2022). Fenugreek seeds contains various bioactive compounds, such as saponins, flavonoids, alkaloids, and polyphenols, that can exert antioxidant, anti-inflammatory, antimicrobial, and immunomodulatory effects (Srinivasa and Naidu, 2021). Fenugreek seeds can also influence the intestinal microbiota by acting as a prebiotic, which is a substrate that can selectively stimulate the growth and/or activity of beneficial bacteria (Zemzmi et al., 2020).

Another potential strategy is the use of direct fed microbials (**DFM**), which are feed additives containing live or viable microorganisms that can confer beneficial effects to the host animal (Dersjant-Li et al., 2013). Among the various types of DFMs, *Bacillus spp*. have been widely used in poultry production due to their ability to produce spores that can withstand harsh conditions, such as high temperature, low pH, and digestive enzymes (Ramlucken et al., 2020). *Bacillus spp.* can modulate the intestinal microbiota by producing antimicrobial substances, enzymes, and organic acids, as well as competing with pathogens for nutrients and adhesion sites (Grant et al., 2018). *Bacillus spp.* can also enhance the immunological response of broilers by stimulating the innate and adaptive immunity, such as toll-like receptors, cytokines, immunoglobulins, and lymphocytes (Dong et al., 2020). *Bifidobacterium* spp. enhanced the productivity, health status, and microbial counts of newly hatched one-day-old broilers (Ahmed et al., 2023).

However, the effects of combining FSs and *Bacillus*-based DFMs on the expression of immune-related genes and cecal microbiome of broilers are not well understood. Therefore, this study was conducted to investigate the effects of selected *Bacillus*-based DFMs on the immune-related gene expression and cecal microbiome in broiler chickens and to correlate these effects with changes in various parameters of intestinal immunity that are associated with protection against infection by enteric pathogens. We hypothesized that the combination of *Bacillus*-based DFMs and FSs would have additive or synergistic effects on immune response and cecal microbial composition of broilers compared to the individual effects of each strategy.

## MATERIALS AND METHODS

### Experimental Design

A total of 160 one-day-old Ross 308 broiler chicks were randomly assigned to four dietary treatment groups, with 5 replicates of 8 birds each, as described in our previous study (Paneru et al., 2023). Birds were raised in floor pens for 42 d and provided with ad libitum access to feed and water. The dietary treatments consisted of control diet (**C**): basal diet formulated based on breeder recommendations for broiler chickens; FS5: control diet supplemented with 5 g/kg of fenugreek seeds; FS5DFM: control diet supplemented with 5 g/kg of fenugreek seeds and 0.1 g/kg of a multi-strain *Bacillus*-based direct-fed microbials containing *B. subtilis, B. amyloliquefaciens*, and *B. licheniformis* (total spore count: 3 × 10^11^ spores/g); and DFM: control diet supplemented with 0.1 g/kg of the same multi-strain *Bacillus*-based direct-fed microbials.

On d 42, one bird was randomly selected from each replicate pen (i.e., 5 birds per dietary treatment) and humanely euthanized via rapid decapitation technique followed by exsanguination. Following euthanasia, approximately 5 cm of the mid-section of ileum between Meckel's diverticulum and ileo-cecal junction was collected and cecal contents were carefully collected from both ceca. Ileum was chosen for gene expression analysis as it is an important site for nutrient absorption and immune function in poultry. Both tissue and cecal contents were immediately submerged in liquid nitrogen for snap-freezing and stored at -80°C until further analysis.

### RNA Isolation and Real-Time Quantitative PCR

Total RNA was extracted from ileum tissues using the PureLink RNA Micro Kit (Invitrogen, Carlsbad, CA) with on-column DNase digestion according to manufacturer's instructions. All work surfaces and equipment were decontaminated with RNaseZap (Invitrogen) solution to prevent RNase contamination. RNA quantity and purity were assessed using a NanoDrop 8000 Spectrophotometer (Thermo Scientific, Waltham, MA). RNA integrity was evaluated by gel electrophoresis using a 2% agarose E-Gel (Thermo Fisher Scientific, Carlsbad, CA). Two micrograms (μg) of pure RNA were reverse transcribed to cDNA using the Maxima First Strand cDNA Synthesis Kit (Thermo Fisher Scientific, Waltham, MA) following the manufacturer's protocol. Oligonucleotide primers specific for chicken immune related genes were designed based on the NCBI gene database using Primer3Plus software (Table 1). β-actin (**ACTB**) was used as the housekeeping gene for normalization and internal control.

**Table 1:** Primer sequences and NCBI accession numbers for selected genes

Gene	Primer sequence (5′ → 3′)	Accession number
Housekeeping gene		
ACTB	F: ACTCTGTCTGGATTGGAGGC	NM_205518.2
	R: AAAGCCATGCCAATCTCGTC	
Avian beta defensins		
AvBD9	F: GTCAGGCATCTTCACAGCTG	NM_001001611.3
	R: GGCTAGGACTTCTCTGTGCA	
AvBD10	F: CACGTCCTGTTAGCACACTG	NM_001001609.3
	R: AGCTGCATGAACCCAAAGTG	
AvBD11	F: CCCTCCTTCAGTTTCCCCTT	NM_001001779.1
	R: CATCTGACTCACTGCTGCAC	
Interleukins		
IL6	F: TGGAAGAAGCATGGAGAGCA	NM_204628.1
	R: GCATCCGTTCCTATGTGCTG	
IL8L2	F: CCGGATATGCAAACACTGGC	NM_205498.1
	R: AGAATTGAGCTGAGCCCTGT	
Caspase		
CASP6	F: CGTGTTCAGTTGGACAGCAA	NM_204726.1
	R: GGAGGGTGCAAAACTGAAGG	
Cyclooxygenase		
PTGS2	F: AAAGGGGCCAGTACTGTGTT	NM_001167718.1
	R: TGCCCAGACTTGTCTTCCTT	
IRF family		
IRF7	F: CACATGTTCATGCTGCTGGA	NM_205372.1
	R: CTGGAAGGAGAGCAGGTTGA	

Real-time PCR was performed using the QuantStudio 6 Pro Real-Time PCR System (Applied Biosystems, Waltham, MA). In a 20 μL reaction volume, 10 ng of cDNA template was amplified with SYBR Green Master Mix (Thermo Fisher Scientific, Waltham, MA) and 400 nM each of forward and reverse primers for each target gene. The thermal cycling conditions for the RT-qPCR were: 50°C for 2 min, 95°C for 10 min, followed by 40 cycles of 95°C for 15 s and 58°C for 1 min. The relative gene expression levels were calculated using the 2^−ΔΔCt^ method with the control group as the calibrator and normalized to the expression of ACTB (housekeeping gene).

### Microbial DNA Extraction and 16s rRNA Sequencing

Cecal content samples stored at -80°C were thawed and processed for DNA extraction using the QIAamp DNA Stool Mini Kit (Qiagen, Hilden, Germany), following the manufacturer's protocol. The concentration and purity of extracted DNA were assessed using a NanoDrop One spectrophotometer (Thermo Fisher Scientific, Madison, WI). The V3-V4 region of the 16S rRNA gene was targeted for amplification due to its conserved nature and ability to discriminate between bacterial taxa. Specific primers (5′-GTGCCAGCMGCCGCGGTAA-3′) and (5′-GGACTACHVGGGTWTCTAAT-3′) were used, alongside Phusion High-Fidelity PCR Master Mix (New England Biolabs Inc., MA) for accurate and efficient amplification. PCR amplicons within the expected size range (400-450 bp) were purified using the Qiagen Gel Extraction Kit (Qiagen, Hilden, Germany) to remove primer dimers and other PCR artifacts. Subsequently, the NEBNext UltraTM DNA Library Prep Kit for Illumina (New England Biolabs Inc., MA, USA) was employed to prepare sequencing libraries with compatible adapters for Illumina sequencing platforms.

Paired-end sequencing reads were processed through a standard bioinformatics pipeline. Briefly, FLASH software (V1.2.7) merged overlapping reads, QIIME (V1.7.0) performed quality filtering and chimera detection using the UCHIME method against the SILVA reference database (Edgar et al., 2011). Operational taxonomic units (**OTU**) were defined at 97% sequence similarity using Uparse software (v7.0.1090), and sequences were taxonomically annotated against the SILVA SSUrRNA database within QIIME. Alpha diversity indices (Shannon, Simpson, ACE, and Chao) were calculated using QIIME to evaluate the within-sample microbial diversity and richness. Beta diversity metrics (weighted and unweighted UniFrac distances) were also computed in QIIME to compare the community composition between samples. Principal component analysis (**PCA**) was performed using the FactoMineR package in R software (Version 2.15.3) to reduce the dimensionality of the beta diversity data and visualize potential clustering patterns among samples.

### Statistical Analysis

One bird from each replicate pen was selected as the experimental unit for gene expression and microbiome analysis. To compare the means of gene expression among different dietary groups, a one-way analysis of variance (**ANOVA**) was performed using SPSS Statistics V28.0 software (IBM Corp., Armonk, NY). Following a significant ANOVA result, Duncan's multiple-range test was used to evaluate the specific pairwise differences between group means. Statistical significance was declared for p-values less than 0.05 and *p*-values between 0.05 and 0.10 were considered trends. The results of the main analyses were presented as mean values with pooled standard error of mean.

Additional statistical analysis, Metastat, was conducted using R software. The LEfSe studies were performed using the LEfSe program. All these indices in our samples were calculated with QIIME (Version 1.7.0). The *p*-values were computed through permutation testing, providing a measure of statistical significance. In parallel, q-values were calculated employing the Benjamini and Hochberg False Discovery Rate method, which aids in controlling the rate of false positives in multiple hypothesis testing (Benjamini and Hochberg, 2000). For the identification of statistical significance among 4 groups, Linear Discriminant Analysis Effect Size (**LEfSe**) analysis was performed using the LEfSe software.

## RESULTS

### Relative Expression of Selected Interleukins in the Ileum of Broilers

**Figure 1:**
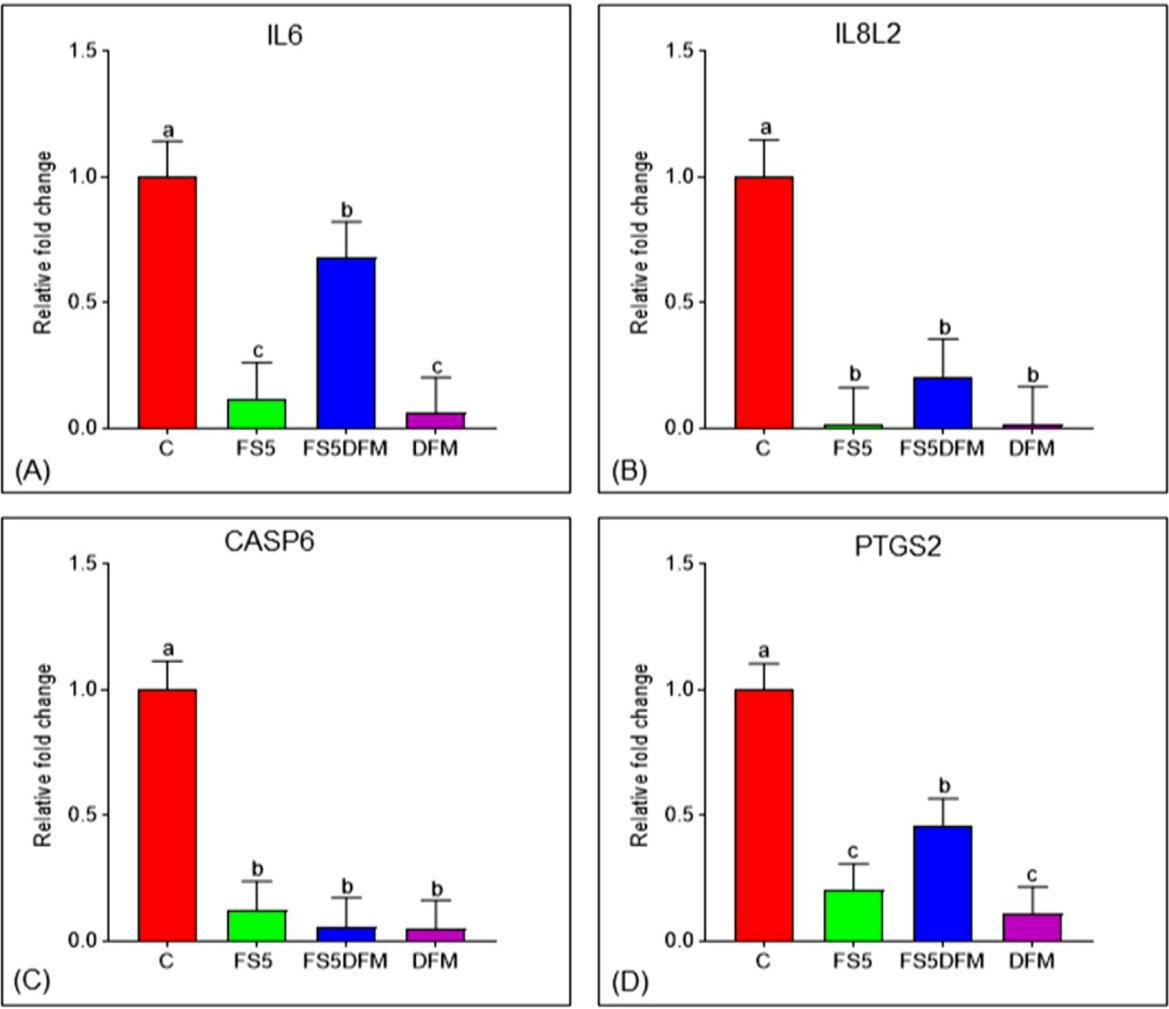
Impact of fenugreek and DFM on immunomodulation and apoptosis gene expression in broiler. Relative expression of IL6 (A), IL8L2 (B), CASP6 (C), and PTGS2 (D) genes in the ileum fed with fenugreek seeds and DFM. Data relates to 5 birds/group (one bird per replicate) and is presented as mean with SEM. Abbreviations: C = Control; FS5 = 5 g/kg inclusion level of fenugreek seed powder; FS5+DFM = 5 g/kg of fenugreek seed powder + 0.1g/kg of Bacillus-based direct-fed microbials; DFM = 0.1g/kg of Bacillus-based direct-fed microbials.

The relative mRNA expression of ileal pro-inflammatory interleukins IL-6 and IL-8L2 in response to dietary inclusion of fenugreek seeds (FS5) and multi-strain *Bacillus*-based direct-fed microbials (**DFM**) in broilers is presented in Figures 1A and 1B, respectively. Both IL-6 and IL-8L2 mRNA levels were significantly downregulated (*P* < 0.001) by FS5, DFM and their combination compared to the control diet (C). Notably, IL-6 expression was significantly higher in the combined DFMFS5 group compared to either treatment alone.

### Relative Expression of Caspase in the Ileum of Broilers

The relative expression of the ileal caspase-6 (**CASP6**) gene following dietary inclusion of fenugreek seeds (**FS5**) and multi-strain *Bacillus*-based direct-fed microbials (**DFM**) in broilers is depicted in Figure 1C. Our results revealed a significant downregulation (*P* < 0.001) of CASP6 expression in broilers fed diets containing either FS5, DFM, or their combination (**FS5DFM**) compared to the control group.

### Relative Expression of Selected Cyclooxygenase in the Ileum of Broilers

The effect of dietary inclusion of fenugreek seeds (FS5) and multi-strain *Bacillus*-based direct-fed microbials (**DFM**) on the ileal prostaglandin-endoperoxide synthase 2 (**PTGS2**) gene expression in broilers is shown in Figure 1D. Both FS5 and DFM supplementation significantly downregulated (*P* < 0.001) PTGS2 mRNA levels compared to the control diet (C). Interestingly, the combined FS5DFM group exhibited a significantly higher expression of PTGS2 compared to individual FS5 or DFM treatment groups.

### Relative Expression of IRF7 in the Ileum of Broilers

**Figure 2:**
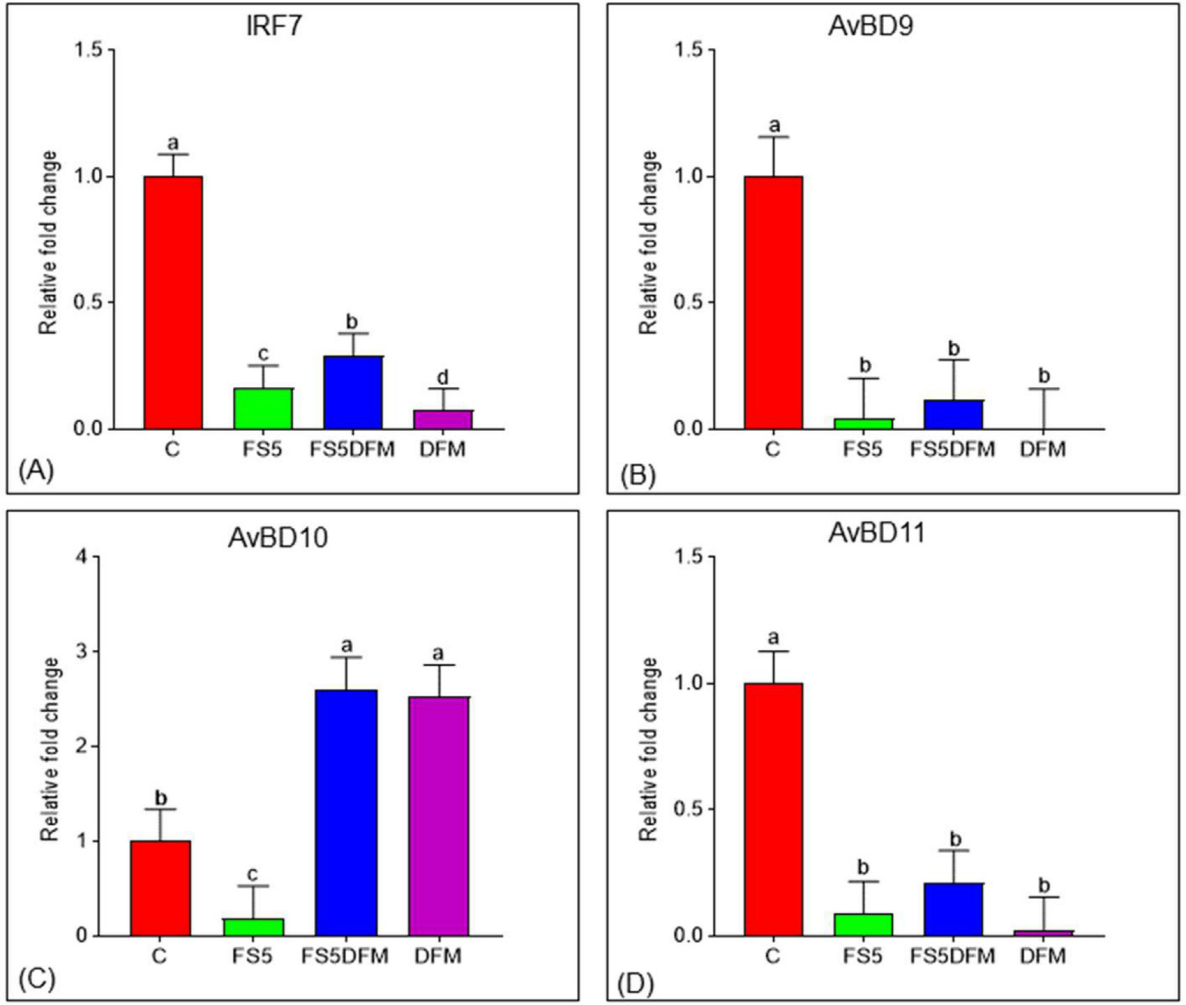
Effects of fenugreek and DFM on antiviral and antimicrobial responses in broiler. Relative expression of IRF7, AvBD9, AvBD10 and AvBD11 genes in the ileum of broiler chickens fed with fenugreek seed and DFM. Data relates to 5 birds/group (one bird per replicate) and is presented as mean with SEM. Abbreviations: C = Control; FS5 = 5 g/kg inclusion level of fenugreek seed powder; FS5DFM = 5 g/kg of fenugreek seed powder + 0.1g/kg of Bacillus-based direct-fed microbials; DFM = 0.1g/kg of Bacillus-based direct-fed microbials.

The relative expression of the interferon regulatory factor 7 (**IRF7**) gene in the ileum of broilers following dietary supplementation with fenugreek seeds (**FS5**) and multi-strain Bacillus-based direct-fed microbials (**DFM**) is shown in Figure 2A. Dietary inclusion of FS5, DFM and their combination significantly downregulated (*P* < 0.001) ileal IRF7 mRNA levels compared to the control group. Interestingly, the expression of IRF7 exhibited lowest expression with the DFM, followed by the FS5 group and the combination group.

### Relative Expression of Selected Avian β-Defensins in the Ileum of Broilers

The effect of dietary inclusion of fenugreek seeds (**FS5**) and multi-strain *Bacillus*-based direct-fed microbials (**DFM**) on the relative expression of avian β-defensins (AvBD9, AvBD10, and AvBD11) in the ileum of broilers is presented in Figures 2B, 2C, and 2D, respectively. Dietary supplementation with either FS5 or DFM significantly impacted the expression of all 3 AvBD genes compared to the control group. AvBD9 and AvBD11 expression exhibited downregulation (*P* = 0.001 and *P* < 0.001, respectively) in response to both FS5 and DFM individually or in combination. Conversely, AvBD10 expression displayed a distinct pattern: it was significantly increased (*P* < 0.001) with DFM and the FS5+DFM combination but decreased with FS5 alone compared to the control.

### Differential Abundance of Microbial Taxa

**Figure 3:**
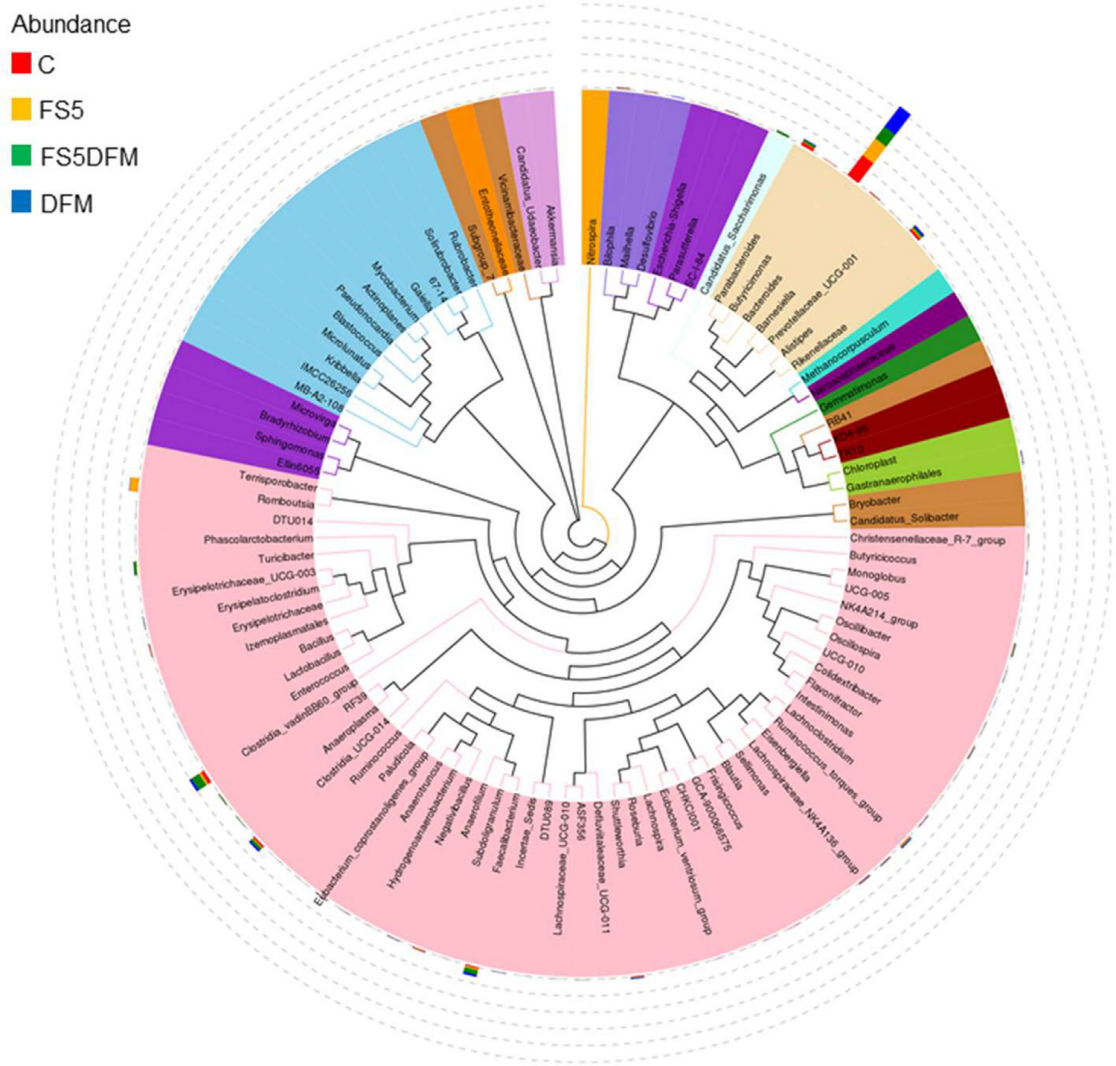
Effects of fenugreek seed and DFM on the gut microbiome diversity of broiler. Data relates to 5 birds/group (1 bird per replicate) and is presented as mean with SEM. Abbreviations: C = Control; FS5 = 5 g/kg inclusion level of fenugreek seed powder; FS5DFM = 5 g/kg of fenugreek seed powder + 0.1g/kg of Bacillus-based direct-fed microbials; DFM = 0.1g/kg of Bacillus-based direct-fed microbials.

Utilizing both a circular cladogram and a linear phylogenetic representation, our comprehensive investigation has identified distinct microbial abundance trends among four distinct treatment groups (FS5, FS5DFM, and DFM) (Figure 3). The circular cladogram details the evolutionary connections, pinpointing taxonomic groups that are evolutionarily close, while the linear phylogenetic diagram offers an intricate view of each taxon's abundance across the groups.

**Figure 4:**
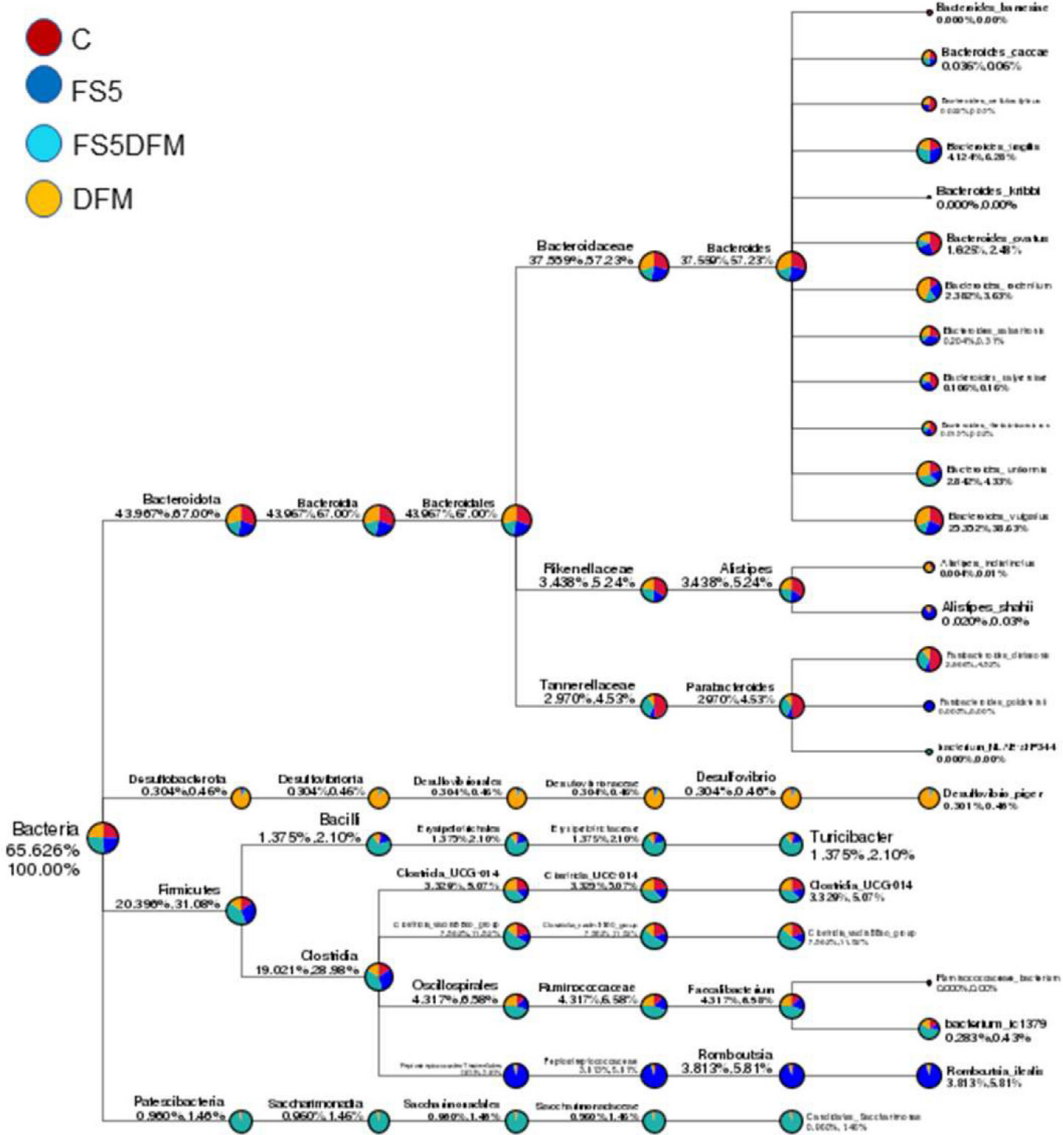
The taxonomy tree of the gut microbiome in the cecal contents of broiler chickens fed with fenugreek seed, DFM, and their combination. Data relates to 5 birds/group (1 bird per replicate) and is presented as mean with SEM. Abbreviations: C = Control; FS5 = 5 g/kg inclusion level of fenugreek seed powder; FS5DFM = 5 g/kg of fenugreek seed powder + 0.1g/kg of Bacillus-based direct-fed microbials; DFM = 0.1g/kg of Bacillus-based direct-fed microbials.

Predominantly, the *Bacteroidetes* and *Firmicutes* phyla are the most abundant within the community, as reflected by their substantial representation. The linear diagram uses colored circles to signify the abundance of taxa; for instance, ‘Bacteroides’ appears consistently across all groups, whereas other taxa are more sporadically distributed, suggesting that certain treatments may either encourage or inhibit the growth of specific microbes (Figure 4). The observed variability in abundance highlights the distinct impact of each treatment on the microbial communities.

### Alpha and Beta Diversity

**Figure 5:**
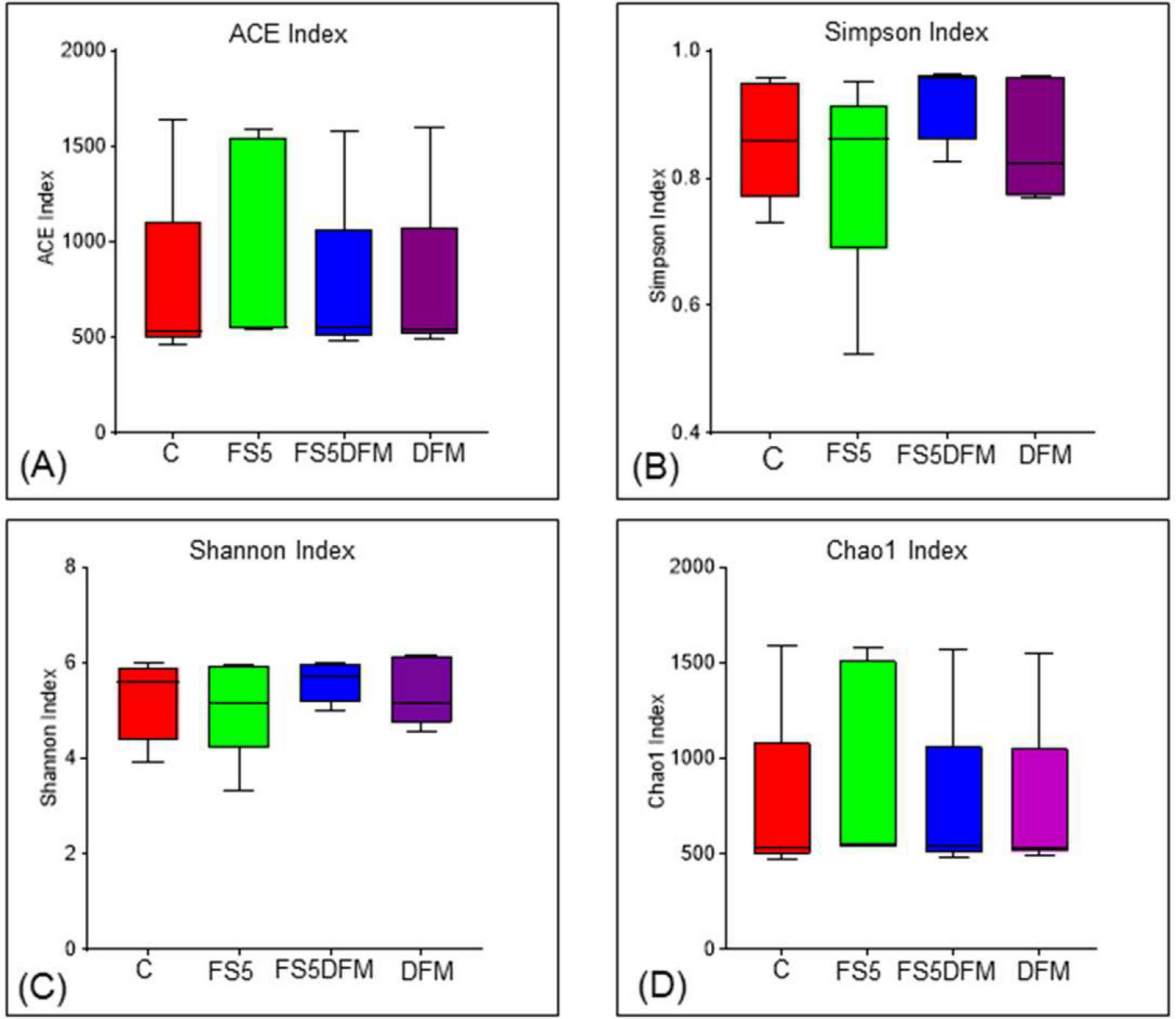
Microbial alpha diversity in the cecal contents of broilers fed with fenugreek seed, DFM, and their combination. Alpha diversity indices: ACE Index (A), Simpson index (B), Shannon index (C), and Chao Index (D); Data relates to 5 birds/group (1 bird per replicate) and is presented as mean with SEM. Abbreviations: C = Control; FS5 = 5 g/kg inclusion level of fenugreek seed powder; FS5DFM = 5 g/kg of fenugreek seed powder + 0.1g/kg of Bacillus-based direct-fed microbials; DFM = 0.1g/kg of Bacillus-based direct-fed microbials.

Statistical analysis of the alpha diversity indices, namely ACE, Simpson, Shannon, and Chao1, was conducted to evaluate the richness and evenness of the gut microbiome across four treatment groups including C, FS5, FS5DFM and DFM (Figure 5). The results indicated no significant differences between the groups for any of the diversity indices used. These results suggest that the alpha diversity of the gut microbiome remained consistent regardless of the treatment applied.

**Figure 6:**
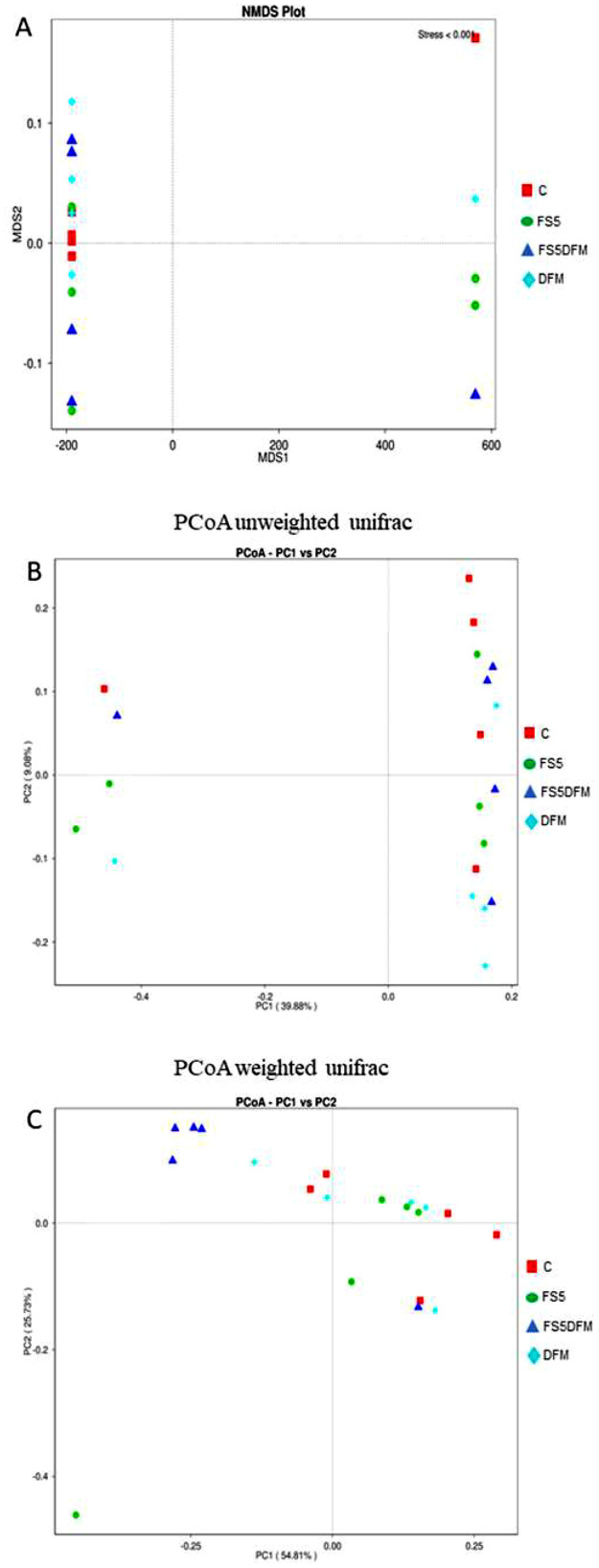
Microbial beta diversity in the cecal contents of broilers fed with fenugreek seed, DFM, and their combination. Beta diversity indices: NMDS plot (A), Weighted Unifrac plot (B), and Unweighted UniFrac Plot (C). Data relates to 5 birds/group (1 bird per replicate) and is presented as mean with SEM. Abbreviations: C = Control; FS5 = 5 g/kg inclusion level of fenugreek seed powder; FS5DFM = 5 g/kg of fenugreek seed powder + 0.1g/kg of Bacillus-based direct-fed microbials; DFM = 0.1g/kg of Bacillus-based direct-fed microbials.

In the beta-diversity analysis depicted in the Non-metric Multidimensional Scaling (**NMDS**) plot (A) indicates a minimal degree of separation among the microbial communities of the four treatment groups C, FS5, FS5DFM, and DFM, suggesting similar community structures across treatments (Figure 6). The Principal Coordinates Analysis (**PCoA**) employing unweighted UniFrac distances (B) reveals subtle clustering, with treatment group FS5DFM showing some divergence from the others, hinting at unique microbial community. In contrast, the PCoA using weighted UniFrac distances (C) demonstrates a more pronounced separation of combination group FS5DFM from the others. This separation is indicative of distinct community structures when the relative abundance of taxa is considered, underlining the unique impact of combination group FS5DFM on microbial composition in terms of both diversity and abundance of species present.

### Characterization of Unique Microbiota

**Figure 7:**
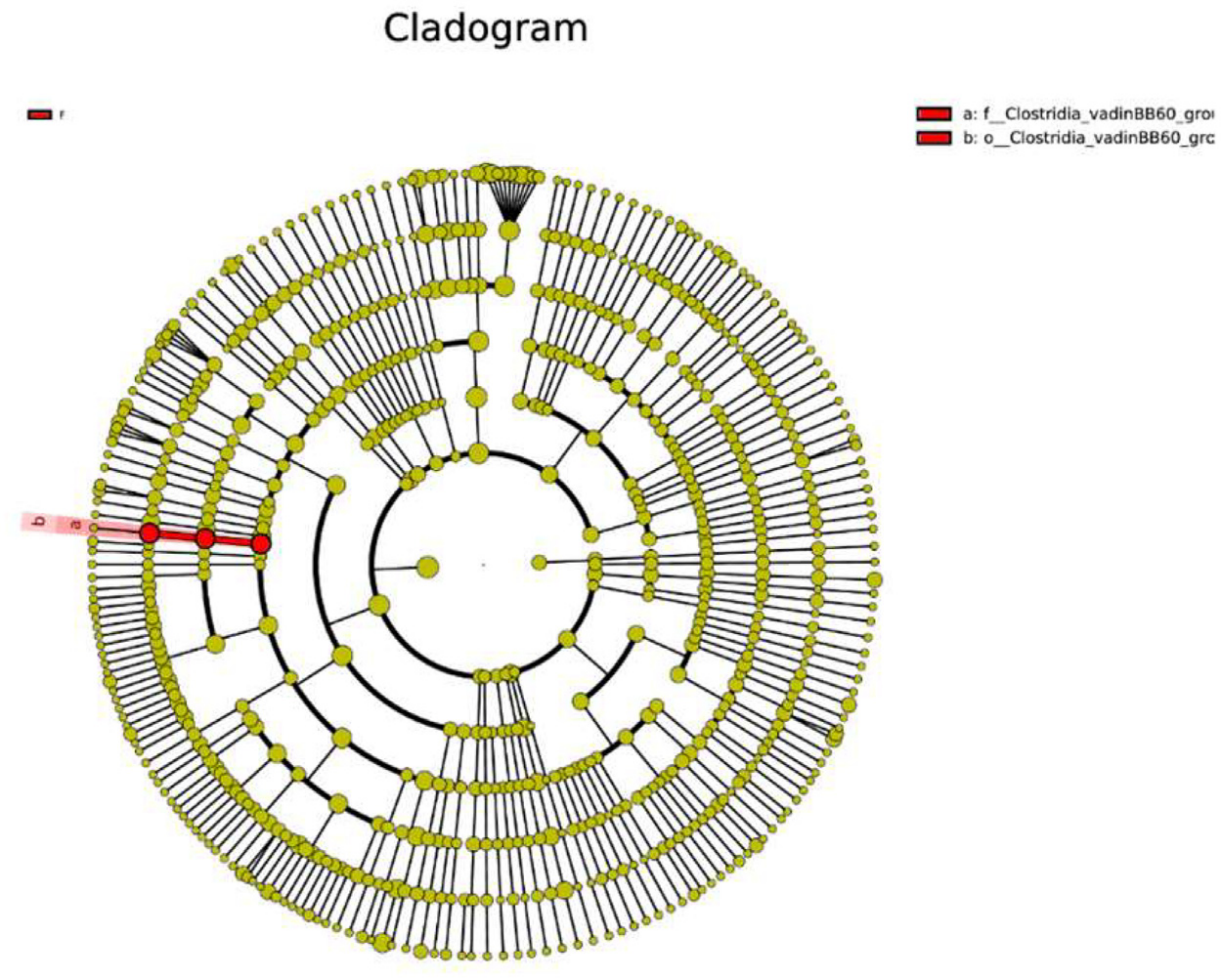
The LEfSe-generated cladogram depicts the bacterial communities in the cecal contents of broilers fed with fenugreek seed, DFM, and their combination. Data relates to 5 birds/group (1 bird per replicate) and is presented as mean with SEM. Abbreviations: C = Control; FS5 = 5 g/kg inclusion level of fenugreek seed powder; FS5DFM = 5 g/kg of fenugreek seed powder + 0.1g/kg of Bacillus-based direct-fed microbials; DFM = 0.1g/kg of Bacillus-based direct-fed microbials.

**Figure 8:**
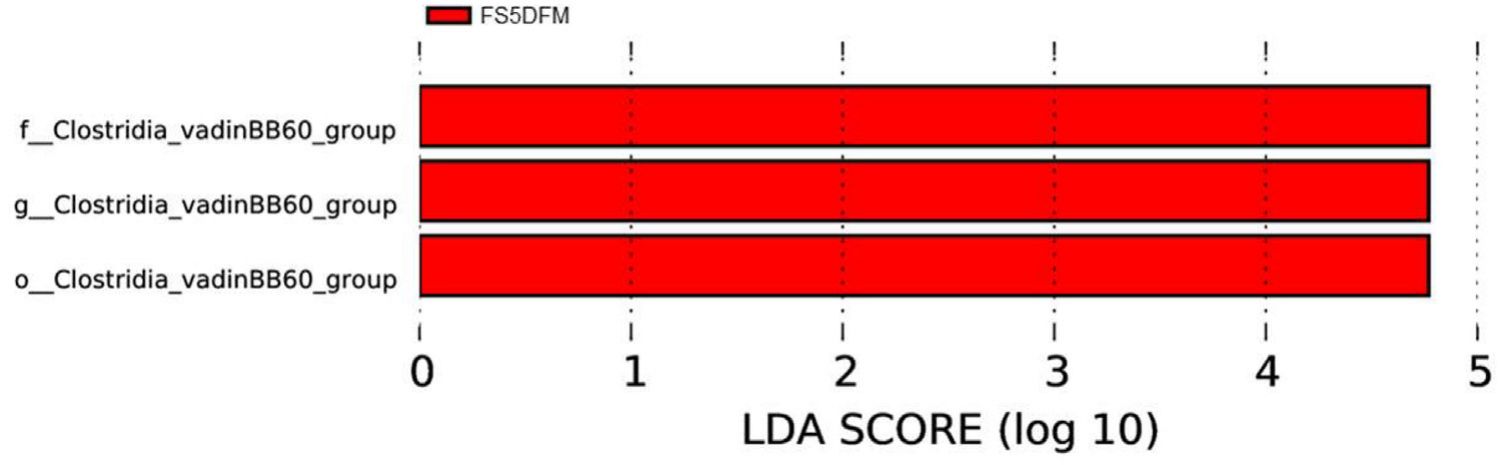
A histogram illustrates the distribution based on linear discriminant analysis (LDA) values in the cecal contents of broilers fed with fenugreek seed, DFM, and their combination. Data relates to 5 birds/group (1 bird per replicate) and is presented as mean with SEM. Abbreviations: C = Control; FS5 = 5 g/kg inclusion level of fenugreek seed powder; FS5DFM = 5 g/kg of fenugreek seed powder + 0.1g/kg of Bacillus-based direct-fed microbials; DFM = 0.1g/kg of Bacillus-based direct-fed microbials.

Employing LEfSe for the comparative analysis of microbial compositions among four distinct groups has unveiled unique bacterial populations characteristic to each respective group (Figure 7). The findings demonstrated that combination group FS5DFM significantly influenced the *Clostridia*_vadinBB60 group at various hierarchical taxonomic stages, with the most pronounced impact being at the family level. The cladogram's configuration indicated that combination group FS5DFM influence is lineage-specific within the *Clostridia*_vadinBB60 group, rather than being uniformly distributed (Figure 8).

## DISCUSSION

A strong and complex relationship exists between the development of a diverse and stable intestinal microbiota and resistance against enteric pathogens (Khan et al., 2021). The composition and functional diversity of the gut microbiota are crucial for preventing enteric infections (Ducarmon et al., 2019). This occurs through competitive exclusion, where beneficial commensals occupy niches and limit the availability of resources for potential pathogens, both exogenous and opportunistic indigenous residents (Modi et al., 2014). Furthermore, resistance against colonization by pathogens may partly result from improvement of the immune system. Additional supplementation of phytogenic in the feed has shown to be beneficial to the gut microbiota suggesting the reduction in the total coliform count while increasing the *Lactobacillus spp.* count (Murugesan et al., 2015). Phytogenics and probiotics represent a promising strategy to enhance the productive performance of broiler chickens by boosting their immune system, optimizing gut microflora composition, and promoting overall health and growth naturally (Abdel-Wareth et al., 2019, 2022; Amer et al., 2022, 2023; Abdel-Wareth and Lohakare, 2023). In this work, phytogenic used was fenugreek seeds. Fenugreek seeds contain 32% insoluble and 13·3% soluble fiber, and have high concentrations of galactose and mannose, which might potentiate a beneficial effect on gut microbiome (Roberts et al., 2011). Our previous study also suggested that 5 g/kg dosage of fenugreek seed has a potential to improve immunity and alter the gut microbiome in broilers (Paneru et al., 2024).

In addition, including direct fed microbials (**DFM**) into the gastrointestinal tract (**GIT**) is a widely adopted practice in the poultry industry to prevent and control enteric infectious diseases (McReynolds et al., 2009; Lee et al., 2010; Tellez et al., 2012). This work aimed to progress further previous findings on broiler performance, blood biochemicals, and intestinal histomorphology (Paneru et al., 2023) and focus on the effects of fenugreek seeds and direct-fed microbials combination on broiler gut microbiota and expressions intestinal immunity related genes that are associated with protection against infection by enteric pathogens. Previous studies with 0.1 g/kg dosage of multi-strain *Bacillus*-based direct-fed microbials has shown efficacy in mitigating necrotic enteritis and *Salmonella* infection in broiler chickens (Adhikari et al., 2019; Hernandez-Patlan et al., 2019).

Our study demonstrated a significant downregulation of the IL-6 and IL8L2 genes in response to both fenugreek seeds and multi-strain *Bacillus* direct-fed microbials. Interestingly, the combination of FSs and DFMs, while still downregulating these genes, exhibited a less pronounced effect compared to either treatment alone. The downregulation of IL-6 and IL8L2 is generally associated with an anti-inflammatory response (Laptev et al., 2019). Moreover, IL-6 and IL8L2 upregulation in chickens has been associated with *Salmonella* and *Eimeria* infection (Lynagh et al., 2000; Laptev et al., 2019). IL-6, a cytokine primarily secreted by T cells and macrophages, exhibits a dual nature, acting as both a pro-inflammatory mediator in the production of acute-phase proteins and an anti-inflammatory cytokine (Scheller et al., 2011). Therefore, the observed downregulation of IL-6 and IL8L2 in our study suggests that both FSs and DFMs exert anti-inflammatory effects in the gut.

Avian β-defensin genes encode the cationic peptides in chickens, and are found in many tissues, including the epithelium of the epidermis, digestive, respiratory, and urogenital tracts of birds (van Dijk et al., 2008). Beta-defensins play a vital role in the integration of innate and acquired immune responses, in addition to their direct antibacterial actions (Menendez and Finlay, 2007). The avian beta-defensin 9 (**AvBD9**) plays a crucial role in the homeostasis of gastrointestinal microbiota and the intestinal immune system (Van Dijk et al., 2023). Some β- defensin genes are expressed all the time in some tissues, and their expression can be upregulated in others in response to microbial infection or pro-inflammatory stimuli (Ramasamy et al., 2012). In other studies, AvBD10 was upregulated in response to *Salmonella* infection in the broiler chicken (Garcia et al.,2021). Evidence suggests that the pathogenic process initiated by a bacterial infection stimulates the expression of several genes in poultry, including those involved in the synthesis of avian β-defensins (AvBD9, AvBD10, AvBD11) (Hong et al., 2012). Our study indicated significant downregulation of AvBD9 and AvBD11 with the inclusion of either FSs alone or DFMs alone and their combination. Therefore, the downregulation of AvBD9 and AvBD11 indicates the absence of infection and shows that FSs and DFMs might reduce the infection caused by pathogens. On the other hand, DFMs and the combination of FSs and DFMs led to a less pronounced upregulation of AvBD10. In this regard, further study of AvBD10 gene expression is warranted with the inclusion of DFMs.

Interferons (**IFN**) are critical factors in fighting viral infections, and they constitute the first line of animal and human defense during infection (Chen et al., 2019). Studies have found significant upregulation of caspase (**CASP6**), prostaglandin-endoperoxide synthase-2 (**PTGS2**), and interferon regulatory factor 7 (**IRF7**) with the pathological process initiated by a bacterial or viral infection (Chen et al., 2019; Laptev et al., 2019). The results of our experiment also indicated significant downregulation of CASP6, PTGS2, and IRF7 with the inclusion of either FSs alone or DFMs alone and their combination compared to the control group. Therefore, the downregulation of the studied immune-related genes with the inclusion of fenugreek seeds and DFMs indicates the absence of infection related to pathogen or less robust immunological response owing to absence of challenge in this study.

Furthermore, the addition of fenugreek seeds and multi-strain *Bacillus* direct-fed microbials, either individually or in combination, had a minimal impact on gut microbiome diversity as measured by alpha diversity indices. While FSs numerically increased some alpha diversity metrics, the differences compared to DFMs, and the combination group were not statistically significant. This suggests that neither treatment significantly altered the overall richness or evenness of the gut microbial community. Similarly, beta-diversity analysis, which assesses the composition and structure of microbial communities, revealed minimal separation among the treatment groups. However, weighted UniFrac, a metric sensitive to phylogenetic relationships, did show some degree of separation between the combination group and the other groups. This suggests that while the overall diversity may not be significantly affected, the combined treatment might influence the specific composition of gut microbes, potentially through additive effects of FSs and DFMs. Furthermore, LEfSe analysis identified a distinct bacterial taxon, *Clostridiales vadin* BB60, exclusive to the combination group. The *Clostridiales vadin* BB60 group is largely unclassified and uncharacterized in its role in metabolism. However, some of them studies have identified these bacteria as short chain fatty acid producing bacteria (Sawicka-Smiarowska et al., 2021; Liu et al., 2023). Supplementation of fenugreek seeds in combination with probiotics improved animal performance primarily by enhancing immunity and gut microflora (Abdel-Wareth et al., 2021). Overall, these findings indicate that both FSs and DFMs individually or in combination exert positive influence on gut immunity, their impact on gut microbiome diversity appears to be subtle.

## CONCLUSIONS

These findings suggest that both FSs and DFMs have the potential to enhance broiler immunity by reducing inflammation. Combining FSs and DFMs may produce synergistic effects on immune and microbial modulation, indicating a need for further investigation using pathogen challenge models. This study indicates that FSs and DFMs individually benefit broiler gut immunity by potentially mitigating inflammation and infection susceptibility. While their impact on overall gut microbiome diversity seems modest, the combination of FSs and DFMs might influence specific bacterial taxa, necessitating additional research. Conducting further studies with a pathogen challenge model could yield valuable insights into the observed synergistic potential and potential antagonistic interactions.

## DISCLOSURES

The authors declare no conflicts of interest.

## References

[bib0007] Abdel-Wareth A.A.A., Hammad S., Khalaphallah R., Salem W.M., Lohakare J. (2019). Synbiotic as eco-friendly feed additive in diets of chickens under hot climatic conditions. Poult. Sci..

[bib0001] Abdel-Wareth A.A.A., Elkhateeb F.S.O., Ismail Zienhom S.H., Ghazalah A.A., Lohakare J. (2021). Combined effects of fenugreek seeds and probiotics on growth performance, nutrient digestibility, carcass criteria, and serum hormones in growing rabbits. Livestock Sci..

[bib0006] Abdel-Wareth A.A.A., Mobashar M., Shah A., Sadiq A. (2022). Jojoba seed oil as feed additive for sustainable broiler meat production under hot climatic conditions. Animals.

[bib0003] Abdel-Wareth A.A.A., Lohakare J. (2023). Bioactive lipid compounds as eco-friendly agents in the diets of broiler chicks for sustainable production and health status. Vet. Sci..

[bib0008] Adhikari B.A., Hernandez-Patlan D., Solis-Cruz B., Kwon YoungMin K.Y., Arreguin M.A., Latorre J.D., Hernandez-Velasco X., Hargis B.M., Tellez-Isaias G. (2019). Evaluation of the antimicrobial and anti-inflammatory properties of Bacillus-DFM (norum tm) in broiler chickens infected with Salmonella Enteritidis. Front. Vet. Sci..

[bib0002] Ahmed M.M.N., Ismail Z.S.H., Elwardany I., Lohakare J., Abdel-Wareth A.A.A. (2023). *In Ovo* feeding techniques of green nanoparticles of silver and probiotics: evaluation of performance, physiological, and microbiological responses of hatched one-day-old broiler chicks. Animals.

[bib0005] Amer S.A., Abdel-Wareth A.A.A., Gouda A., Saleh G.K., Nassar A.H., Sherief W.R.I.A., Albogami S., Shalaby S.I., Abdelazim A.M., Abomughaid M.M. (2022). Impact of dietary lavender essential oil on the growth and fatty acid profile of breast muscles, antioxidant activity, and inflammatory responses in broiler chickens. Antioxidants.

[bib0004] Amer S.A., Mahmoud F., Gouda A., Abdel-Wareth A.A.A., Abdel-Warith A.A., Younis E.M., Elshopakey G.E., Baher W.M., Saleh G.K., Davies S.J. (2023). New Insights into the effects of microbial muramidase addition in the diets of broiler chickens. Animals.

[bib0009] Benjamini Y., Hochberg Y. (2000). On the adaptive control of the false discovery rate in multiple testing with independent statistics. J. Educ. Behav. Stat..

[bib0010] Blake D.P., Knox J., Dehaeck B., Huntington B., Rathinam T., Ravipati V., Ayoade S., Gilbert W., Adebambo A.O., Jatau I.D., Raman M. (2020). Re-calculating the cost of coccidiosis in chickens. Vet. Res..

[bib0011] Bortoluzzi C., Vieira B.S., Hofacre C., Applegate T.J. (2019). Effect of different challenge models to induce necrotic enteritis on the growth performance and intestinal microbiota of broiler chickens. Poult. Sci..

[bib0013] Chen S., Wang T., Liu P., Yang C., Wang M., Jia R., Zhu D., Liu M., Yang Q., Wu Y., Zhao X. (2019). Duck interferon regulatory factor 7 (IRF7) can control duck Tembusu virus (DTMUV) infection by triggering type I interferon production and its signal transduction pathway. Cytokine.

[bib0014] Choi J., Goo D., Sharma M.K., Ko H., Liu G., Paneru D., Choppa V.S.R., Lee J., Kim W.K. (2023). Effects of different Eimeria inoculation doses on growth performance, daily feed intake, gut health, gut microbiota, foot pad dermatitis, and Eimeria gene expression in broilers raised in floor pens for 35 days. Animals.

[bib0015] Clark B., Panzone L.A., Stewart G.B., Kyriazakis I., Niemi J.K., Latvala T., Tranter R., Jones P., Frewer L.J. (2019). Consumer attitudes towards production diseases in intensive production systems. PloS one.

[bib0016] Dersjant-Li Y., Awati A., Kromm C., Evans C. (2013). A direct fed microbial containing a combination of three-strain Bacillus sp. can be used as an alternative to feed antibiotic growth promoters in broiler production. J. Appl. Anim. Nutr..

[bib0017] Dong Y., Li R., Liu Y., Ma L., Zha J., Qiao X., Chai T., Wu B. (2020). Benefit of dietary supplementation with Bacillus subtilis BYS2 on growth performance, immune response, and disease resistance of broilers. Probiotics Antimicrob. Proteins..

[bib0018] Ducarmon Q.R., Zwittink R.D., Hornung B.V.H., Van Schaik W., Young V.B., Kuijper E.J. (2019). Gut microbiota and colonization resistance against bacterial enteric infection. Microbiol. Mol. Biol. Rev..

[bib0019] Dziva F., Stevens M.P. (2008). Colibacillosis in poultry: unravelling the molecular basis of virulence of avian pathogenic Escherichia coli in their natural hosts. Avian Pathol.

[bib0020] Ebrahimi-Nik H., Bassami M.R., Mohri M., Rad M., Khan M.I. (2018). Bacterial ghost of avian pathogenic E. coli (APEC) serotype O78: K80 as a homologous vaccine against avian colibacillosis. PLoS One.

[bib0021] Edgar R.C., Haas B.J., Clemente J.C., Quince C., Knight R. (2011). UCHIME improves sensitivity and speed of chimera detection. Bioinformatics.

[bib0022] Emami N.K., Dalloul R.A. (2021). Centennial review: recent developments in host-pathogen interactions during necrotic enteritis in poultry. Poult. Sci..

[bib0023] Garcia J.S., Byrd J.A., Wong E.A. (2021). Tissue-, age-and dose-dependent changes in avian β-defensin and LEAP2 mRNA abundance in the intestines of Salmonella Typhimurium challenged broilers. Anim. Biotechnol..

[bib0024] Grant A., Gay C.G., Lillehoj H.S. (2018). Bacillus spp . as direct-fed microbial antibiotic alternatives to enhance growth, immunity, and gut health in poultry. Avian Pathol.

[bib0025] Hernandez-Patlan D., Solis-Cruz B., Pontin K.P., Hernandez-Velasco X., Merino-Guzman R., Adhikari B., López-Arellano R., Kwon Y.M., Hargis B.M., Arreguin-Nava M.A., Tellez-Isaias G. (2019). Impact of a Bacillus direct-fed microbial on growth performance, intestinal barrier integrity, necrotic enteritis lesions, and ileal microbiota in broiler chickens using a laboratory challenge model. Front. Vet. Sci..

[bib0026] Hong Y.H., Song W., Lee S.H., Lillehoj H.S. (2012). Differential gene expression profiles of β-defensins in the crop, intestine, and spleen using a necrotic enteritis model in 2 commercial broiler chicken lines. Poult Sci.

[bib0027] Khan I., Bai Y., Zha L., Ullah N., Ullah H., Shah S.R.H., Sun H., Zhang C. (2021). Mechanism of the gut microbiota colonization resistance and enteric pathogen infection. Front. Cell. Infect. Microbiol..

[bib0028] Laptev G.Y., Filippova V.A., Kochish I.I., Yildirim E.A., Ilina L.A., Dubrovin A.V., Brazhnik E.A., Novikova N.I., Novikova O.B., Dmitrieva M.E., Smolensky V.I. (2019). Examination of the expression of immunity genes and bacterial profiles in the caecum of growing chickens infected with Salmonella Enteritidis and fed a phytobiotic. Animals.

[bib0029] Lee K., Lillehoj H.S., Siragusa G.R. (2010). Direct-fed microbials and their impact on the intestinal microflora and immune system of chickens. J. Poult. Sci..

[bib0030] Liu H., Li X., Zhang K., Lv X., Zhang Q., Chen P., Wang Y., Zhao J. (2023). Integrated multi-omics reveals the beneficial role of chlorogenic acid in improving the growth performance and immune function of immunologically stressed broilers. Anim. Nutr..

[bib0031] Lynagh G.R., Bailey M., Kaiser P. (2000). Interleukin-6 is produced during both murine and avian Eimeria infections. Vet. Immunol. Immunopathol..

[bib0032] McReynolds J., Waneck C., Byrd J., Genovese K., Duke S., Nisbet D. (2009). Efficacy of multistrain direct-fed microbial and phytogenetic products in reducing necrotic enteritis in commercial broilers. Poult Sci.

[bib0033] Menendez A., Finlay B.B. (2007). Defensins in the immunology of bacterial infections. Curr. Opin. Immunol..

[bib0034] Modi S.R., Collins J.J., Relman D.A. (2014). Antibiotics and the gut microbiota. J. Clin. Invest..

[bib0035] Mohebodini H., Jazi V., Bakhshalinejad R., Shabani A., Ashayerizadeh A. (2019). Effect of dietary resveratrol supplementation on growth performance, immune response, serum biochemical indices, cecal microflora, and intestinal morphology of broiler chickens challenged with Escherichia coli. Livest. Sci..

[bib0036] M'Sadeq S.A., Wu S., Swick R.A., Choct M. (2015). Towards the control of necrotic enteritis in broiler chickens with in-feed antibiotics phasing-out worldwide. Anim. Nutr..

[bib0037] Murugesan G.R., Syed B., Haldar S., Pender C. (2015). Phytogenic feed additives as an alternative to antibiotic growth promoters in broiler chickens. Front. Vet. Sci..

[bib0038] Paneru D., Tellez-Isaias G., Arreguin-Nava M.A., Romano N., Bottje W.G., Asiamah E., Abdel-Wareth A.A., Lohakare J. (2023). Effect of fenugreek seeds and Bacillus-based direct-fed microbials on the growth performance, blood biochemicals, and intestinal histomorphology of broiler chickens. Front. Vet. Sci..

[bib0039] Paneru D., Tellez-Isaias G., Bottje W.G., Asiamah E., Abdel-Wareth A.A., Salahuddin M., Lohakare J. (2024). Modulation of immune response and cecal microbiota by dietary fenugreek seeds in broilers. Vet. Sci..

[bib0040] Ramasamy K.T., Verma P., Reddy M.R. (2012). Differential gene expression of antimicrobial peptides β defensins in the gastrointestinal tract of Salmonella serovar Pullorum infected broiler chickens. Vet. Res. Commun..

[bib0041] Ramlucken U., Lalloo R., Roets Y., Moonsamy G., Van Rensburg C.J., Thantsha M.S. (2020). Advantages of Bacillus-based probiotics in poultry production. Livest. Sci..

[bib0042] Roberts K.T. (2011). The potential of fenugreek (Trigonella foenum-graecum) as a functional food and nutraceutical and its effects on glycemia and lipidemia. J. Med. Food..

[bib0043] Ruwali P., Pandey N., Jindal K., Singh R.V. (2022). Fenugreek (Trigonella foenum-graecum): Nutraceutical values, phytochemical, ethnomedicinal and pharmacological overview. South Afr. J. Bot..

[bib0044] Sawicka-Smiarowska E., Bondarczuk K., Bauer W., Niemira M., Szalkowska A., Raczkowska J., Kwasniewski M., Tarasiuk E., Dubatowka M., Lapinska M., Szpakowicz M. (2021). Gut microbiome in chronic coronary syndrome patients. J. Clin. Med..

[bib0045] Scheller J., Chalaris A., Schmidt-Arras D., Rose-John S. (2011). The pro- and anti-inflammatory properties of the cytokine interleukin-6. Biochim. Biophys. Acta BBA - Mol. Cell Res..

[bib0046] Shahbandeh, M. 2023. Estimated animal protein consumption worldwide in 2022, by source (in million metric tons). Statista.

[bib0047] Srinivasa U.M., Naidu M.M., Atta-ur- Rahman (2021). Studies in Natural Products Chemistry, 71.

[bib0048] Tellez G., Pixley C., Wolfenden R.E., Layton S.L., Hargis B.M. (2012). Probiotics/direct fed microbials for Salmonella control in poultry. Food Res. Int..

[bib0049] van Dijk A., Veldhuizen E.J., Haagsman H.P. (2008). Avian defensins. Vet. Immunol. Immunopathol..

[bib0050] van Dijk A., Guabiraba R., Bailleul G., Schouler C., Haagsman H.P., Lalmanach A.C. (2023). Evolutionary diversification of defensins and cathelicidins in birds and primates. Mol. Immunol..

[bib0051] Zemzmi J., Ródenas L., Blas E., Najar T., Pascual J.J. (2020). Characterisation and in vitro evaluation of fenugreek (Trigonella foenum-graecum) seed gum as a potential prebiotic in growing rabbit nutrition. Animals.

